# *Didymocarpus lobulatus* (Gesneriaceae), a new species from Zhejiang Province, East China

**DOI:** 10.3897/phytokeys.157.30349

**Published:** 2020-08-26

**Authors:** Wen-Yuan Xie, Jia-Jun Zhou, Xin Hong, Fang Wen

**Affiliations:** 1 Monitoring Center for Forest Resources of Zhejiang, CN-310020, Hangzhou, China Monitoring Center for Forest Resources of Zhejiang Hangzhou China; 2 Guangxi Key Laboratory of Plant Conservation and Restoration Ecology in Karst Terrain, Guangxi Institute of Botany, Guangxi Zhuangzu Autonomous Region and Chinese Academy of Sciences, CN-541006 Guilin, China Anhui University Hefei China; 3 School of Resources and Environmental Engineering, Anhui University, CN–230601, Hefei, China, Guangxi Zhuangzu Autonomous Region and Chinese Academy of Sciences Guilin China; 4 Gesneriad Conservation Centre of China, Guilin Botanical Garden, Guangxi Zhuangzu Autonomous Region and Chinese Academy of Sciences, CN-541006 Guilin, China Guangxi Institute of Botany Guilin China

**Keywords:** *Flora of Zhejiang*, new taxon, taxonomy, *Didymocarpus* sect. *Heteroboea*

## Abstract

*Didymocarpus lobulatus*, a new species endemic to Zhejiang province, eastern China, is described and illustrated with photographs. The new species is morphologically similar to *D. heucherifolius*, *D. cortusifolius* and *D. salviiflorus* in leaf morphology, but can be easily distinguished by a combination of characters, including the shape of bracts, calyx and calyx lobes.

## Introduction

The delimitation of *Didymocarpus* has varied considerably over time with recent results from both molecular phylogenetic studies and morphological revisions (Weber et al. 2011, Möller et al. 2011, Möller and Clark 2013, Li et al. 2016). Now, prior to the new species being described here, there are approximately 70 species in the world (Weber et al. 2000) consisting of 34 species and four varieties in China (Cai et al. 2016).

*Didymocarpus* Wall. (Gesneriaceae) was once considered to consist of about 180 species (Wang et al. 1998). However, it was split into three genera: *Didymocarpus* s.s., *Henckelia* Spreng., and *Hovanella* A.Weber & B.L.Burtt (Weber and Burtt 1997). Thus, *Didymocarpus* was left with about 70 species after the removal of the Madagascan, southern Indian and Sri Lankan and most Malesian species (Nangngam and Maxwell 2013). *Didymocarpus* sensu stricto currently contains 70 species (Vitek et al. 2000, Weber et al. 2000, 2011). According to the treatment of *Didymocarpus* s.l. in Flora of China (Wang et al. 1990, 1998), this genus was recognised and divided into two sections: sect. Didymocarpus (herbs with stems (0.7–)3–62 cm long) and sect. Heteroboea (herbs stemless). The first section, Didymocarpus sect. Didymocarpus, is a natural unit with about 50 species distributed mainly in Bhutan, Burma, Southwest China, Malaya, Nepal, North & Northeast India, North Thailand and North Vietnam. Some new taxa belonging to this section were discovered and published recently, e.g. *Didymocarpus puhoatensis* Xin Hong & F. Wen, (2018), *D. moellerii* A.Joe, Hareesh & M.Sabu, (2016), *D. anningensis* Y.M. Shui, Lei Cai & J. Cai, (2016), *D. tonghaiensis* J.M. Li & F.S. Wang, (2015). The other section, sect. Heteroboea, includes seven species and one variety and is endemic to South and East China (Wang et al. 1990, 1998; Li and Wang 2004). In the past five years, two new taxa were discovered and published in eastern and southern China, namely Didymocarpus heucherifolius var. yinzhengii J.M. Li & S.J. Li (2014) from Hunan province and *D. dissectus* Fang Wen, Y.L. Qiu, Jie Huang & Y.G. Wei, (2013) from Fujian province.

In 2010, one of the authors (HX) found an unknown *Didymocarpus* species with the previous year’s fruits during field investigations in Zhejiang Province, China. Soon afterwards, the same species was again collected by the other authors (XWY & ZJJ) during floristic surveys in 2014. Based on the recollected specimens of this uncertain species and from detailed inspection in 2016 and 2017, we confirmed it belongs to *Didymocarpus* because of its disc-like stigma (Wang et al. 1998) and it is a member of sect. Heteroboea as it is a stemless herb. We also concluded that this plant is new to science after thoroughly consulting the related literature (Wang et al. 1990, Wang et al. 1998, Li and Wang 2004). Here, the new species is described and illustrated and its morphological characters are compared with its morphologically similar congeners.

## Material and methods

Measurements and morphological character assessments of the putative new species were undertaken and described using available specimens stored in the following herbaria in China, the United States and the United Kingdom: AHU, E, IBK, KUN, MO, PE and US. In addition, images of other type specimens were obtained from Tropicos (http://www.tropicos.org) and JSTOR Global Plants (http://plants.jstor.org). All morphological characters were studied under dissecting microscopes and are described using the terminology presented by Wang et al. (1998).

## Taxonomic treatment

### Didymocarpus lobulatus

Taxon classificationPlantaeLamialesGesneriaceae

F. Wen, Xin Hong & W.Y. Xie sp. nov.

61DAA285-C8F9-556F-AD02-CA8DDDE0E16A

urn:lsid:ipni.org:names:77211191-1

Figures 1 , 2

#### Diagnosis.

*Didymocarpus lobulatus* is similar to *D. heucherifolius* Handel-Mazzetti and *D. salviiflorus* W.Y. Chun in having a similar zygomorphic corolla and pink to pinkish-purple funnel-shaped to tubular corolla tube, but can be distinguished from the former by its densely eglandular and glandular pubescent peduncle (vs. villous in *D. heucherifolius*), bracts subulate to subulate-triangular (vs. elliptic) and margin sparsely crenate from the middle (vs. entire), calyx shallowly 5-lobed to or lobed about two-thirds of the calyx length from the base (vs. 5-lobed to the base). From *D. salviiflorus*, it differs by having subulate to subulate-triangular bracts (vs. semi-orbicular in *D. salviiflorus*), calyx lobes triangular but non-overlapping (vs. depressed oblong, overlapping at margin) and in size in ca. 5 × 2.5 mm (vs. 2–2.2 × 4–4.5 mm). It is also similar to *D. cortusifolius* in the shape and size of the leaves, but can be easily distinguished by having bracts subulate to subulate-triangular (vs. ovate to elliptic in *D. cortusifolius*), bract margin sparsely crenate from the middle (vs. entire), larger calyx lobes ca. 5 × 2.5 mm (vs. 1–3 × ca. 2 mm) and lobes’ margin nearly entire to entire (vs. denticulate), corolla white (vs. corolla pink to dark pink), fertile stamens adnate to corolla ca. 5 mm from base (vs. adnate to corolla 10–14 mm from base), filaments sparsely brownish glandular-puberulous (vs. glabrous).

**Figure 1.**
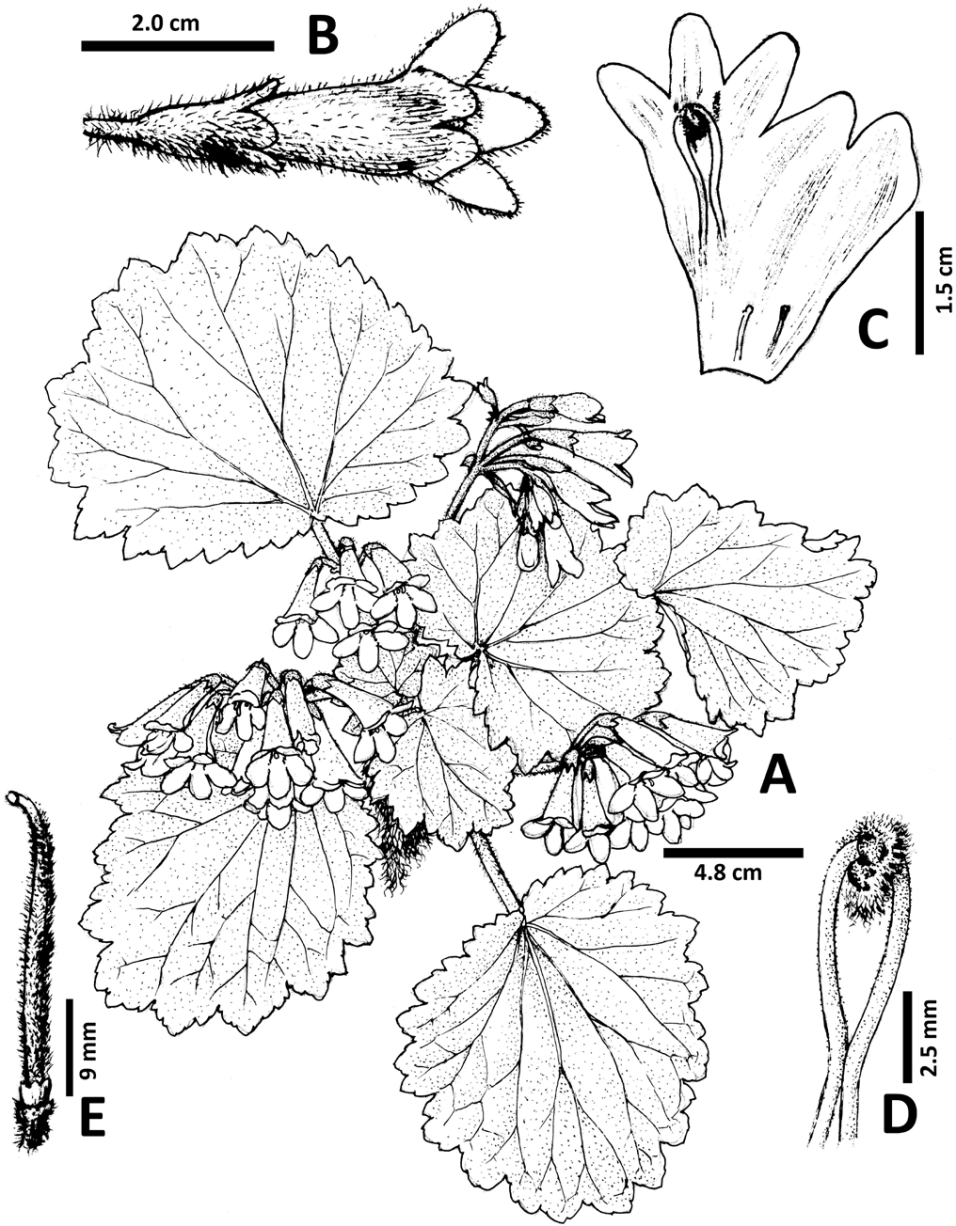
*Didymocarpus lobulatus***A** Habit **B** Flower in top view **C** Opened corolla, showing stamens and staminodes **D** Fertile stamens and anthers **E** Pistil, disc and stigma.

#### Type.

CHINA. Zhejiang Province: Shengzhou city, Chongren Town, Liwang village, 29°39'N, 120°39'E, 223 m a.s.l., 23 May 2014, flowering, Wen-Yuan Xie & Jia-Jun Zhou 140523-01 (holotype: IBK; isotype: AHU).

#### Description.

Perennial acaulescent herb. Rhizome horizontal, 1–4 cm long, up to 1.5 cm in diameter, roots fibrous. Leaves 4–8 basal, clustered at the apex of the rhizome; petioles terete, 2–9 cm long, densely covered with spreading rust-brown villous and white puberulent indumentum; blades asymmetrically orbicular-ovate to orbicular triangular, 3–10 × 2.5–12.5 cm, apex rounded, base cordate, margin irregularly triangular denticulate, papery, upper surface densely covered with whitish short and long eglandular pubescent indumentum, green, lower surface sparsely covered with hairs as on upper surface confined to the veins, pale green, basic veins 3–6, obscure above, prominent beneath. Inflorescences axillary, cymes 1–2-branched, 3–8 (–12)-flowered; peduncle 3–16 cm long, densely covered with both eglandular and glandular pubescent hairs, pedicel 0.3–2 cm long, with indumentum as on the peduncle. Bracts 2, opposite, subulate to subulate-triangular, ca. 8 mm long, adaxially glabrous, abaxially puberulent, margin sparsely crenate from the middle; bracteoles 2, opposite, subulate, 3–3.5 mm long, indumentum same as bracts. Calyx actinomorphic, shallowly 5-lobed to about two-thirds of the calyx length from the base, symmetrical, 1.3–1.6 cm long, inside glabrous, outside densely puberulent, brownish-green; lobes equal, triangular, ca. 5 × 2.5 mm, apices obtuse, margin nearly entire to entire. Corolla zygomorphic, 2.5–3.2 cm long; outside sparsely puberulent to glabrescent, inside glabrous, pink to dark pink, becoming pinkish-white at the base, with brown lines inside. Tube funnel-shaped to tubular, 1.8–2.2 cm long, 0.8–1 cm in diameter at mouth; limb distinctly 2-lipped, adaxial lip 2-parted to near the middle, lobes ovate, ca. 6 × 5 mm, abaxial lip 3-lobed from the base, lobes oblong, ca. 8 × 4 mm, more or less equal. Fertile stamens 2, adnate to corolla ca. 0.5 cm from base; filaments slender, 6–10 mm long, geniculate near the base, sparsely brownish glandular-puberulous; anthers fused along their entire adaxial surfaces, reniform-oblong, 1.8–2.5 mm long, ca. 2 mm wide, pale yellow, bearded on the back; staminodes 3, reduced to capitate, 0.3–0.5 mm long, adnate to corolla 6-8 mm from base, brown. Disc cylindrical, ca. 2 mm long, margin irregular, glabrous. Pistils 2–3 cm long, densely puberulent with both glandular and eglandular hairs; ovary ca. 2.6 cm long, yellowish-green. Stigma 1, terminal, depressed-globose, undivided, translucent. Capsule 5.5–7 cm long, brownish, glabrous.

**Figure 2.**
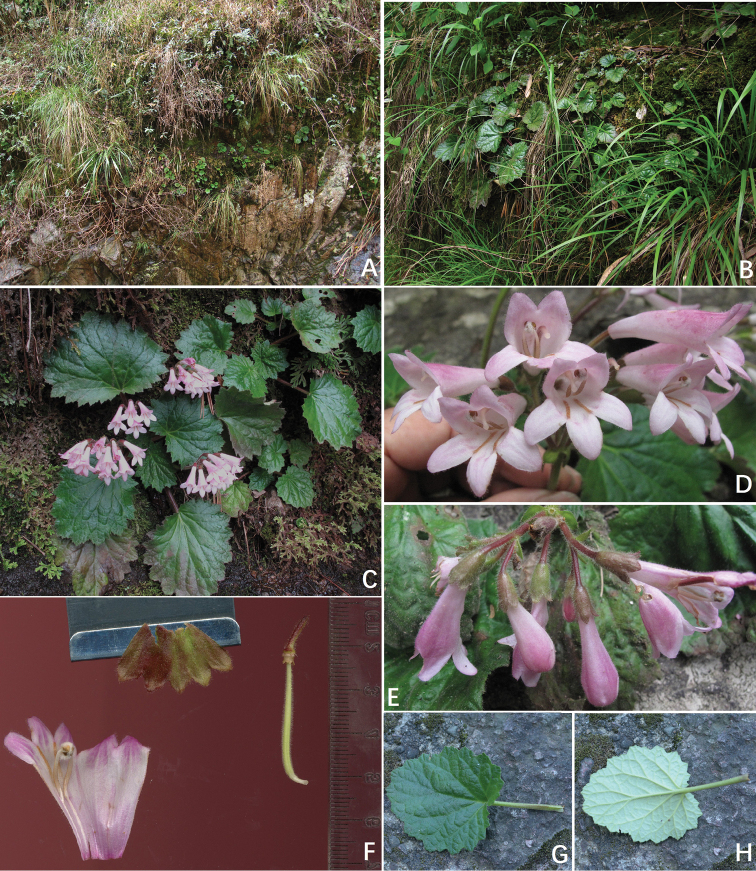
*Didymocarpus lobulatus* F. Wen, Xin Hong & W.Y. Xie. **A** Habitat **B** Vegetative part of plants **C** Habitat in flowering **D** Frontal view of corolla **E** Top view of cyme, showing actinomorphic calyx **F** Opened corolla for showing stamens, pistil and calyx lobes **G** Adaxial surface view of leaf blade **H** Abaxial surface view of leaf blade.

#### Etymology.

The specific epithet is derived from the shallowly 5-lobed calyx.

#### Distribution and habitat.

*Didymocarpus lobulatus* is locally abundant and endemic to a narrow area in eastern China, surrounding the type locality. This species grows on moist shady cliffs of sandy shale hills, at an elevation of 223 m a.s.l. in type locality. The average temperature is 16.4 °C, the average annual precipitation has been calculated as ca. 1,446.8 mm. The forest is a subtropical monsoon climate evergreen broad-leaved forest. Flowering in May. Another population growing in the Danxia landscape of Chuanyanshijiufeng, Xinchang County, Shaoxing city, not far away from the type locality, was discovered by the first author in 2016. The two places are about 40 kilometres apart.

#### Proposed conservation status.

Based on the present field investigations, *Didymocarpus lobulatus* is currently only known from two sites around the type locality. The two places are about 40 kilometres apart. The type population, which grew close to a country road, is potentially threatened by human activities. Although no such habitat destruction is currently occurring, this population is likely to be threatened in the foreseeable future under influences of man-made factors, for example, by road widening. Fortunately, the second population has been found in the well-protected core zone of the scenic spot, Chuanyanshijiufeng. Furthermore, this population is healthy and locally abundant with many young plants and seedlings growing in the area. Thus, it seems that this species is not at serious risk now and it is proposed as ‘Vulnerable’ (VU D2) according to the IUCN Red List Categories and Criteria (IUCN 2016).

#### Notes.

**Map 1.**
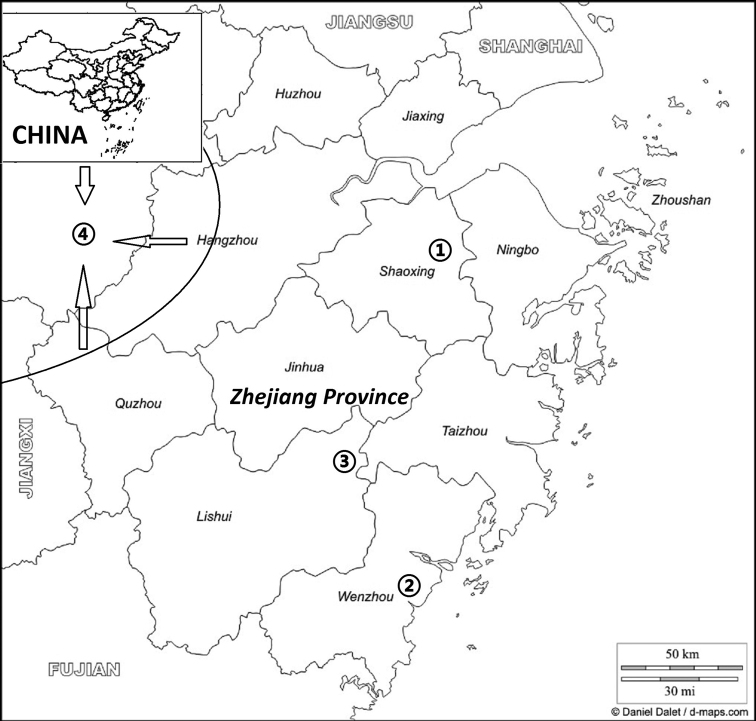
The distribution areas of *Didymocarpus lobulatus* and its congeners in Zhejiang prov.: ➀ *D. lobulatus* ➁ *D. cortusifolius* ➂ *D. salviiflorus* ➃ *D. heucherifolius* (in semi-elliptical region, the arrows point).

**Figure 3.**
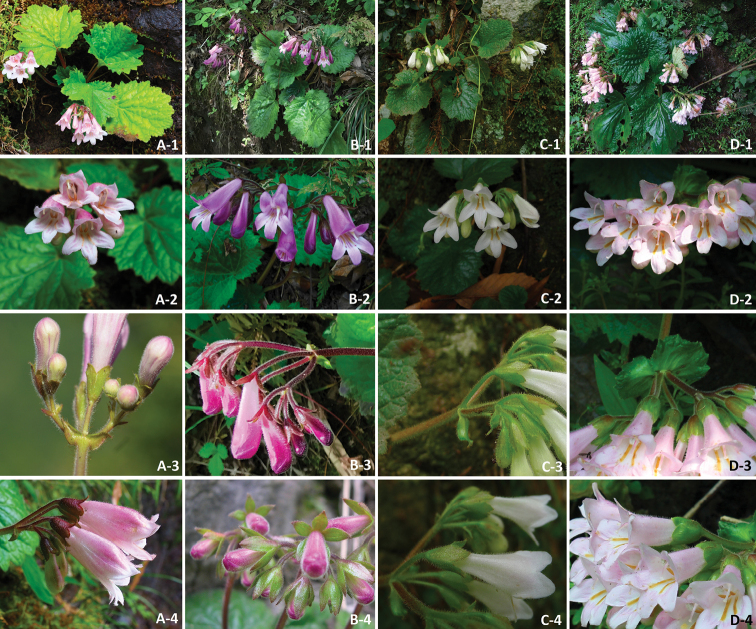
**A***Didymocarpus lobulatus***B***D. heucherifolius***C***D. cortusifolius***D***D. salviiflorus***1** Habitats **2** The frontal view of corolla and cyme **3** Cyme and bracts **4** calyx lobes.

The geographical distributions of *Didymocarpus lobulatus* and its congeners are identified in Map 1. There are altogether 7 species and one variety of sect. Heteroboea, three species are endemic to East China (*Didymocarpus salviiflorus* and *D. cortusifolius* in Zhejiang prov. and *D. dissectus* in Fujian prov.) and two species and one variety (*D. yuenlingensis* W.T. Wang, *D. sinoprimulinus* W.T. Wang and D. heucherifolius var. yinzhengii) endemic to Hunan prov. of South China. Only one species, *Didymocarpus heucherifolius*, is widespread, being distributed from northern Guangdong prov. of South China, to Jiangxi prov., Fujian prov., Anhui prov. and Zhejiang prov. of East China. As more and more field investigations are undertaken, the biodiversity of sect. Heteroboea in East and South China will be better understood by researchers. The discovery of two new taxa, *Didymocarpus dissectus* Fang Wen, Y.L. Qiu, Jie Huang & Y.G. Wei (2013) and *D. lobulatus*, serve as two good examples. Differences between the new species and its morphologically related species in sect. Heteroboea in Zhejiang prov. are shown in the following identification table (Table 1) and Figure 3.

**Table 1.** Diagnostic character differences amongst *Didymocarpus lobulatus* sp. nov., *D. heucherifolius*, *D. cortusifolius* and *D. salviiflorus*.

Character		*D. lobulatus*	*D. heucherifolius*	*D. cortusifolius*	*D. salviiflorus*
**Bracts**	**shape**	subulate to subulate-triangle	elliptic	ovate to elliptic	Semi-orbicular
	**size**	ca. 8 × 2–3 mm	5-10 × 1.8–4.5 mm	3.5–7 × 1.5–3 mm	ca. 5 × 10 mm
	**margin**	sparsely crenate from the middle	entire	entire	sparsely crenate
**Bracteoles**	**shape**	subulate	usually lacking	ovate to oblong	usually lacking
**Calyx**	**form**	actinomorphic, shallowly 5-lobed to about two-thirds of the calyx length from the base	slightly zygomorphic, 5-lobed to the base	actinomorphic, 5-lobed to about half of the calyx length from the base	actinomorphic, 5-lobed to about half of the calyx length from the base
	**lobes size**	ca. 5 × 2.5 mm	3–4 × 1–2 mm	1–3 × ca. 2 mm	2–2.2 × 4–4.5 mm
	**lobes margin**	nearly entire to entire	sparsely denticulate	denticulate	denticulate to entire
**Stamens**	**place**	adnate to corolla ca. 5 mm from base	adnate to corolla 10-12 mm from base	adnate to corolla 10-14 mm from base	adnate to corolla ca. 10 mm from base
	**indumentum**	sparsely brownish glandular-puberulous	white glandular-puberulous	nearly glabrous	sparsely white puberulent

#### Other specimen examined.

CHINA. Zhejiang Province: Shaoxing city, Xinchang County, Chuanyanshijiufeng, Danxia landscape, secondary forests, 29°23'N, 120°48'E, 248 m a.s.l., 11 November 2018, in fruit, *WYG181111-01* (IBK!).

## Supplementary Material

XML Treatment for Didymocarpus lobulatus
